# Use of rituximab as a treatment for systemic lupus erythematosus: retrospective review

**DOI:** 10.1590/S1679-45082014AO2706

**Published:** 2014

**Authors:** Roberta Ismael Lacerda Machado, Morton Aaron Scheinberg, Maria Yvone Carlos Formiga de Queiroz, Danielle Christinne Soares Egypto de Brito, Maria Fernanda Brandao de Resende Guimarães, Raquel Altoé Giovelli, Eutilia Andrade Medeiros Freire

**Affiliations:** 1 Universidade Federal da Paraíba, João Pessoa, PB, Brazil; 2 Hospital Israelita Albert Einstein, São Paulo, SP, Brazil; 3 Universidade Federal do Espírito Santo, Vitória, ES, Brazil

**Keywords:** Lupus erythematosus, systemic/drug therapy, Antibodies, monoclonal/therapeutic use

## Abstract

**Objective:**

To report the experience in three Brazilian institutions with the use of rituximab in patients with different clinical forms of lupus erythematosus systemic in activity.

**Methods:**

The study consisted of a sample of 17 patients with LES, who were already being treated, but that at some stage of the disease showed refractory symptoms. The patients were subdivided into groups according to the clinical manifestation, and the responses for the use of rituximab were rated as complete, partial or no response. Data were collected through a spreadsheet, and used specific parameters for each group. The treatment was carried on by using therapeutic dose of 1g, and repeating the infusion within an interval of 15 days.

**Results:**

The clinical responses to rituximab of the group only hematological and of the group only osteoarticular were complete in all cases. In the renal group there was a clinical complete response, two partial and one absent. In the renal and hematological group complete response, there was one death and a missing response. The pulmonary group presented a complete response and two partial.

**Conclusion:**

The present study demonstrated that rituximab can bring benefits to patients with lupus erythematosus systemic, with good tolerability and mild side effects; it presented, however, variable response according to the system affected.

## RESUMO

**Objetivo:**

Relatar a experiência obtida em três instituições brasileiras com o uso do rituximabe em pacientes com diferentes formas clínicas de lúpus eritematoso sistêmico em atividade.

**Métodos:**

Estudo composto por amostra de 17 pacientes portadores de lúpus, que já faziam tratamento, mas que, em algum momento da evolução da doença, apresentaram sintomas refratários. Os pacientes foram subdivididos em grupos de acordo com o acometimento clínico que motivou o uso do imunobiológico, e a resposta ao uso do rituximabe foi classificada como completa, parcial ou ausente. Os dados foram coletados por meio de uma planilha padronizada, sendo utilizados parâmetros específicos para cada grupo. O tratamento foi padronizado com dose terapêutica de 1g, com repetição da infusão em um intervalo de 15 dias.

**Resultados:**

As respostas clínicas ao rituximabe dos grupos apenas hematológico e do apenas osteoarticular foi completa em todos os casos. No grupo renal, houve uma resposta clínica completa, duas parciais e uma ausente. No grupo renal e hematológico, houve uma resposta completa, um óbito e uma resposta ausente. O grupo pulmonar apresentou um caso de resposta clínica completa e dois parciais.

**Conclusão:**

O presente estudo demonstrou que rituximabe pode trazer benefícios aos pacientes com lúpus eritematoso sistêmico, com tolerabilidade boa e efeitos colaterais brandos, apresentando, contudo, resposta variável, de acordo com o sistema acometido.

## INTRODUCTION

Systemic Lupus Erythematosus (SLE) is a chronic multisystem disease of unknown etiology. It is characterized by inflammation of the connective tissue and by the presence of antinuclear autoantibodies (ANA), particularly double-stranded anti-DNA antibodies (dsDNA), and presents with variable clinical manifestations; it is progressive and potentially fatal if not treated.^(1)^

Autoantibody production contributed towards the development of SLE by induction of immune cells mediated by type III and type II hypersensitivity, and antibody-dependent cytotoxicity. Antibody deposition may instruct the innate immune cells in the production of cytokines such as interferon alpha (IFNα), tumor necrosis factor (TNF), and interleukins (IL).^(2)^

A fundamental immunological aspect of SLE is participation of B-cells in the activity of the disease. Experimentally it has been noted that when these cells were removed from “lupus models” in mice, by gene manipulation or antibody therapy, the chain of immune reactions reaction was largely suppressed, including anomalies induced by T-cells.^(3,4)^ B-cells may regulate other B-cells, T-cells, and dendritic cells, besides producing various cytokines, including IL-4 and IL-10. Despite the fact that involvement of B-lymphocytes in SLE is not yet fully understood, it is known that their participation is fundamental in the immunopathogenesis of this disease.^(5)^

Rituximab(RTX) is a chimeric monoclonal antibody against CD20, an antigen expressed on B-cells that was used for the first time in 1997 to treat non-Hodgkin lymphomas.^(6)^ In 2006, it was approved for the treatment of rheumatoid arthritis, in combination with methotrexate, for patients non-responsive to treatment with anti-TNFagents.^(7)^

The use of RTX in SLE had its first case reported in 2009; since then, it has been used off-label in some specific cases of autoimmune diseases, demonstrating efficacy.^(6)^

## OBJECTIVE

To report the experience obtained with the use of rituximab in patients with systemic lupus erythematosus in three national institutions, and demonstrate, by clinical observation, that its use may constitute a new therapeutic option.

## METHODS

The present study is a retrospective analysis composed of a random sample of 17 patients with SLE, treated at Rheumatology Services in three Brazilian cities: Hospital of the *Universidade Federal da Paraíba*, in João Pessoa (PB); *Hospital Israelita Albert Einstein*, in São Paulo (SP); and at the *Santa Casa de Misericórdia,* in Vitória (ES).

Inclusion criteria used for the selection of the sample were to present with SLE with the diagnosis confirmed as per the criteria of the American College of Rheumatology,1997;^(8)^ an absence or inefficiency of the clinical response to previously used medications to treat SLE; present with adverse events related to the medications previously used, age between 18 and 60 years, under treatment with RTX.

Patients were classified according to the clinical picture that motivated the use of the immunobiological agent, with the formation of the following groups: renal, hematological, osteoarticular, and pulmonary. However, some patients simultaneously fit into more than one group.

Treatment was performed by giving the patients 1g doses of RTX, with repetition of the infusion with a 15 day interval. Data were collected by means of patient chart review, using a data collection card that grouped them at times 0,30, 60, and 90 days after the first infusion.

The dependent variables were hemoglobin, leukocytes, platelets, creatinine, urea, and 24 hour proteinuria. Independent variables were gender, age, and skin color.

Total clinical response was defined as normalization of the clinical and laboratorial parameters. The partial response was defined as an improvement in parameters when these were compared to the initial time, without, however, reaching normality. The absent response was determined when there was no improvement of the patient's clinical picture, worsening, or death after the treatment suggested.

The analysis algorithm of the data was directed by descriptive statistics, using distribution of frequency and measurements of central tendency, such as medians. All variables were analyzed by means of Excel Word 2010, on a standardized spreadsheet.

The project had the support of the National Council of Scientific and Technological Development (CNPq, CAAE: 0027.0.126.000-08).

## RESULTS

The study sample had the participation of 17 patients, 15 of them females. Mean age of patients was 36.21 years, with a standard deviation of 10.77 and a median of 34.5.

The frequency for each group of clinical ailments was three patients with pulmonary involvement, two osteoarticular, five hematologic, four renal, and three with association of hematologic and renal involvement.

Response to RTX, according to organ/system affected, is shown on chart 1.

**Chart 1:** Groups ordered according to organ/system and response to rituximab

Organ/system affected	Clinical involvement	Response to rituximab
Pulmonary	Lupus pneumonitis	Complete
Pulmonary	Pleural serositis	Partial
Pulmonary	Interstitial pneumopathy	Partial
Hematologic	Thrombocytopenia	Complete
Hematologic	Thrombocytopenia	Complete
Hematologic	Thrombocytopenia	Complete
Hematologic	Thrombocytopenia	Complete
Hematologic	Anemia	Complete
Renal	Nephrotic syndrome	Complete
Renal	Membranoproliferative	Partial
Renal	Membranoproliferative	Partial
Renal	Membranoproliferative	Absent
Hematologicand renal	Leukopenia and membranous glomerulonephritis	Absent
Hematologic and renal	Thrombocytopenia, anemia and membranous glomerulonephritis	Complete/late
Hematologic and renal	Hemolytic anemia, thrombocytopenia and membranous glomerulonephritis	Absent/death
Osteoarticular	Severe arthritis	Complete
Osteoarticular	Severe arthritis	Complete

**Figure 1:**
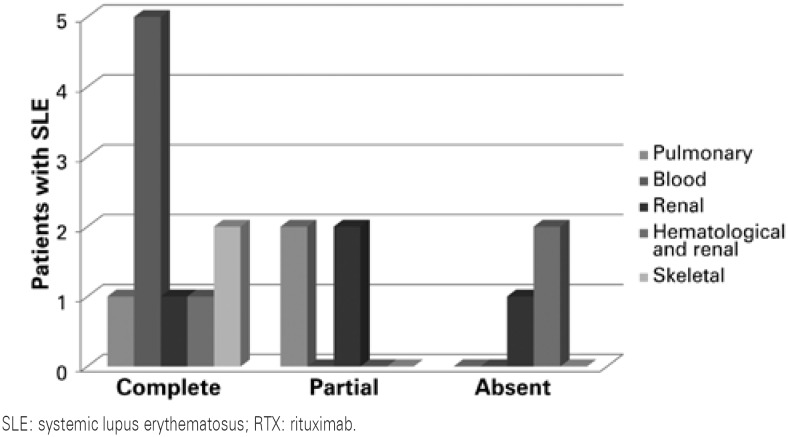
Clinical response to the use of rituximab in patients with systemic lupus erythematosus, according to the affected organ/system

The type of clinical response to treatment was classified as absent, partial, or complete, and is represented on figure 1.

In the pulmonary group, there were three cases: one with lupus pneumonitis with complete response to RTX and the other two with a partial response, one with pleural serositis and the other with interstitial pneumopathy.

The clinical response of the group with only hematologic involvement that progressed with thrombocytopenia and anemia was complete and significant in all cases.

In the group with renal compromise, there were four cases, three of which presented with membranoproliferative type glomerulonephritis, two of them with partial responses and one with an absent response, and one case presented with nephrotic syndrome, responding completely to treatment.

In the renal and hematologic group there were three patients: one with nephritis and leukopenia, one with hemolytic anemia, membranoproliferative nephritis and thrombocytopenia (both had absent responses), and one case with thrombocytopenia, anemia, and nephritis (that presented with a complete late response).

The osteoarticular group had two cases of severe polyarthritis, with significant limitations of mobility in the patients.

There were no reports of side effects related to the medication.

## DISCUSSION

The quantity of medications used in rheumatologic ailments has increased greatly over the last few years. This is attributed to the introduction of specific medications, which act directly on the events of the inflammatory process and involve various molecules, among them, cytokines and cellular receptors, which establishes so-called “biological therapy”.

Treatment with RTX in SLE already has reports of series of cases with satisfactory results.^(9)^ Recent publications have confirmed the role of RTX in treating various systemic manifestations of SLE, with improvement of the clinical parameters or arthritis, serositis, nephritis, hemolytic anemia, and thrombocytopenia, besides the recovery of serum parameters and indicators of disease activity, thus allowing the reduction or suspension of medications such as corticosteroids and other immunosuppressants.^(9,10)^ Despite the fact that there is are not yet regimes of consensual applications and doses, the most often used in clinical practice is the scheme introduced by Edwards et al. for rheumatoid arthritis.^(11)^ In the study done by Vital et al. with 39 patients, RTX proved effective in SLE, and the clinical response was related to the number of B-cells, with predictive outcome variables the depletion of B-lymphocytes and repopulation, using highly sensitive flow cytometry in assessment of these patients.^(12)^

In the present study, among the patients with renal compromise, no substantial clinical improvement was confirmed after treatment with RTX. Nevertheless, van Vollenhoven et al. demonstrated improvement in histological activity in two patients with lupus nephritis refractive to the use of cyclophosphamide who received RTX.^(13)^ The same had been reported earlier by Leandro et al. who described an experience of a reference service with the use of RTX in six patients with SLE and nephritis refractory to conventional treatment, reporting good results in five of them.^(14)^

The depletion of B-lymphocytes may interfere in the production of pathological antibodies against erythrocyte and platelet antigens, which could justify a significant improvement in hematologic involvement in cases of SLE treated with RTX. Many authors have demonstrated this efficacy:^(15-18)^ Landeiro et al. for example, described that all the patients of their study that presented with autoimmune cytopenia were benefited with the use of RTX.^(19)^ In our study, the five patients with hematological manifestations also attained a positive complete response after using RTX.

Despite the small sample, this study was capable of corroborating the results obtained by Scheinberg et al. in a retrospective study with patients presenting with autoimmune diseases refractory to high doses of corticotherapy, but that responded satisfactorily to the use of RTX.^(20)^

Correlating the impact of RTX in manifestations by systems with previous studies, we were able to observe that the two patients with severe arthritis obtained a complete clinical response with the use of this treatment, which was in agreement with the publication by Edward et al.^(11)^. Pulmonary involvement in SLE is rare and corresponds to 3 to 13% of complications; however, the clinical picture is severe and resistant to conventional treatment.^(21)^ Lim et al. described the first case of interstitial pneumonitis in a patient with SLE treated with RTX that obtained improvement of symptoms and of pulmonary function tests, besides decreasing the dose of prednisone in the follow-up of these patients.^(22)^

In the present study, the three patients with pulmonary involvement responded clinically to RTX. Further observed was a case of interstitial pneumonitis that obtained a complete response, besides a case of pneumonitis and another of serositis that obtained partial responses to the treatment.

Martínez-Martínez and Abud-Mendonza reported the case of a young patient with pulmonary involvement, with recurrent diffuse alveolar hemorrhage, that benefited with treatment with RTX.^(23)^ There are various recent studies that evidence results similar to those presented.^(24)^

In this retrospective evaluation, the use of RTX was not associated to adverse effects related to the infusion, due to the pre-treatment performed. The assessment of side effects in patients with SLE who received RTX is made difficult by the possibility of these effects also being caused by the disease itself or by concomitant use of other immunosuppressants.^(25)^

A considerable controversy is shown, however, when randomized studies have not pointed towards superior results with the use of RTX, even when clinical practice shows positive data. Merrill et al. analyzed 257 patients, distributed randomly at a 2:1 ratio, to receive RTX (1,000mg) or placebo on days 1, 15, 168, and 182. The treatment adjuvant to RTX was uniformly distributed among azathioprine, mycophenolate mofetil, and methotrexate. No differences were observed between the placebo and RTX, highlighting only a beneficial effect of RTX on Afro-American and Hispanic subgroups. Safety and tolerability were similar in patients receiving placebo and those receiving RTX.^(26)^

Rovin et al. studied the efficacy and safety of RTX in a randomized, double-blind, placebo-controlled study in patients with lupus nephritis treated concomitantly with mycophenolate and corticosteroids. One hundred and forty-four patients were evaluated, randomized at a 1:1 ratio to receive RTX (1,000mg) or placebo on days 1, 15, 168, and 182. Renal biopsies were performed at Week 52. Although therapy with RTX led to more accentuated reductions in the levels of anti-dsDNA and C3/C4, better clinical results were not seen after one year of treatment. Combinations of RTX with mycophenolate and corticosteroids did not result in any sign of additional safety nor in new unpublished results.^(27)^

A survey of those patients that received RTX for lupus nephritis performed by Díaz-Lagares et al. consisted of 164 patients diagnosed with lupus nephritis confirmed by biopsy, 145 of them women and 19 men, with a mean age of 32.3 years. RTX was administered in combination with corticosteroids in 162 patients, and immunosuppressant agents in 124 patients; of these, 58 were treated with cyclophosphamide and 55 with mycophenolate. In the 6th and 12th months, the rates were, respectively, 27% and 30% for a complete response; 37% and 40% for a partial response; and 36% and 30% for no response. A significant improvement was noted in 24-hour proteinuria (4.41g baseline *versus* 1.31g post-treatment; p=0.006), serum albumin (2.8g at baseline *versus* 3.6g post-treatment; p<0.001), and proteinuria/creatinuria ratio (of 421.94g at baseline/mmol to 234.98g of baseline/mmol post-treatment; p<0.001). A significant improvement of response was found in patients with type III lupusnephritis compared with types IV and V (p=0.007 and 0.03, respectively). Nephrotic syndrome and renal insufficiency, at the time of RTX administration, predicted a worse response (p=0.001 and 0.024, respectively). In this study, RTX proved an effective option for patients with lupus nephritis, especially those refractive to standard treatment or that experienced a new crisis after intensive immunosuppressant treatment.^(28)^

In a recent editorial, Scheinberg discusses the controversy between the results obtained by randomized studies as to the use of RTX in lupus and clinical practice, suggesting that, instead of a failure of medication, it could be due to a flaw in protocol design. He pointed out that heterogeneity of forms of presentation of SLE, its gravity, and the influence of ethnicity hinder the description of clinical studies, thus impeding the viability of studies with larger samples and statistical significance for new therapies. Additionally, many of these studies exclude individuals with more serious renal and central nervous system manifestations, in order to not expose these patients to possible risks.^(29)^

### Study limitations

The primary limitation of this study is found in its retrospective model. Nevertheless, one should consider that it contains a reasonable number of cases and that it provides a view of the clinical practice use of this therapy in SLE, as well as a literature review, suggesting that RTX may benefit patients with SLE, with a good level of tolerability and mild side effects.

## CONCLUSION

The authors intended, with disclosing these results, to contribute towards the use of effective treatments for patients with systemic lupus erythematosus refractory to the use of conventional medications, seeking new treatment alternatives that might guarantee better quality of life to these patients.
